# Selenium and the risk of cancer in BRCA1 carriers

**DOI:** 10.1186/1897-4287-9-S2-A5

**Published:** 2011-06-01

**Authors:** J Lubinski, K Jaworska, K Durda, A Jakubowska, T Huzarski, T Byrski, M Stawicka, J Gronwald, B Górski, W Wasowicz, E Kilar, M Szwiec, D Surdyka, E Marczyk, P Sun, SA Narod

**Affiliations:** 1 Department of Genetics and Pathology, International Hereditary Cancer Center, Pomeranian Medical University, Szczecin, Poland; 2 Prophylactic and Epidemiology Center, Poznan, Poland; 3 Institute of Occupational Medicine, Lodz, Poland; 4 Regional Oncology Hospital, Swidnica, Poland; 5 Regional Oncology Hospital, Opole, Poland; 6 Center of Oncology of Lublin Region, Lublin, Poland; 7 Regional Oncology Center, Krakow, Poland; 8 Womens College Research Institute and Dalla Lana School of Public Health, University of Toronto, Canada

## 

It has not been established if dietary factors or nutritional supplements impact on the incidence of cancer in high-risk women. We randomised 1135 women with a BRCA1 mutation to 250 micrograms daily of elemental selenium as sodium selenite, or to placebo, in a double-blind trial. After a median follow-up period of 35 months (range 6 to 62 months), there were 60 incident cases of cancer diagnosed in the selenium-supplemented group, versus 45 cases in the placebo group (hazard ratio 1.4; 95% CI: 0.9 to 2.0). Selenium supplementation was not associated with a reduction in the risk of primary breast cancer (hazard ratio 1.3; 95% CI: 0.7 to 2.5), of contralateral breast cancer (hazard ratio 1.5; 95% CI: 0.7 to 3.2), or of ovarian cancer (hazard ratio 1.3; 95% CI: 0.6 to 2.7). The results of this study do not support the recommendation that selenium supplementation should be offered to BRCA1 carriers for chemoprevention.

Part II Adnexectomy, genotypes and selenium level as markers of the risk of cancer

In these part we conducted a nested case-control study of 68 women with breast cancer and 17 women with ovarian cancer and 170 controls matched 1 to 2. Cases and controls were matched for age at enrolment, past history of breast cancer, oophorectomy and whether they received selenium supplement or placebo during cancer chemoprevention trial. Combinations of clinical status, genotypes and selenium levels strongly associated with extremely low risk of cancer have been identified.

The strongest associations have been found for GPX4 variants:

a. all nTT and Se level 60-80µg/l – OR 0.32, p: 0.0009

b. all TT and Se level >80µg/l – OR 0.10, p: 0.047

c. for carriers without adnexectomy and with TT variant, Se level >80µg/l – OR 0.038, p: 0.014.

